# Investigation of the thermal performance of a solar absorber and nocturnal radiator (SAANR) hybrid panel for summer and winter seasons

**DOI:** 10.1016/j.heliyon.2020.e05764

**Published:** 2020-12-21

**Authors:** Kago R. Rabasoma, Kevin N. Nwaigwe

**Affiliations:** Department of Mechanical Engineering, University of Botswana, Gaborone, Botswana

**Keywords:** Nocturnal cooling, Diurnal heating, Hybrid, Transient, Thermal performance, Temperature

## Abstract

Transient analysis and performance prediction of a solar absorber and nocturnal radiator (SAANR) hybrid panel for water heating and cooling in the summer and winter seasons of Gaborone, Botswana is presented. Transient heat transfer models were developed for the panel and the models were then transformed into explicit finite difference forms for numerical analysis using MATLAB. The SAANR panel added up to 7 kWh diurnal heating energy in winter and removed up to 0.5 kWh at night through radiation cooling in summer. The resulting numerical solutions of the model were validated by comparison with experimental results and they showed excellent strength of association with a correlation coefficient of 0.832 and fairly acceptable goodness of fit at an R-squared value of 69%. Sensitivity analysis carried out showed the model to react linearly to panel area changes. From the results, it is concluded that the hybrid panel is robust enough to perform in both the summer and winter seasons of Gaborone, and areas with similar climatic conditions. This implies that the system can be used simultaneously for space cooling and domestic water heating in both summer and winter. This technology can therefore be used instead of electric water heaters and air-conditioners and thus save energy.

## 1 Introduction

Increasing mobility requirements and rising electricity needs for buildings represent a huge increase in global energy demand currently, and it is projected to further increase over the next decades. Concerns about responsible energy use are well documented in the literature and focuses on the costs of space conditioning, thus intensive research on energy-saving space conditioning technologies is essential. In many countries, building energy consumption accounts for approximately 40% of total energy demands, and of that fraction, the energy required for space heating and cooling of buildings is about 60%, which accounts for the largest percentage of energy usage [1, 2, 3]. The latest developments in energy research have been concentrated more on harnessing the ambient environment towards space conditioning. Night sky cooling has been applied throughout time in all its complexities and innovations for space cooling [4]. The use of a nocturnal radiator has been found to yield the best possible results of radiative cooling and that it can be used in conjunction with a solar water heater as a hybrid system to serve two purposes of space cooling and domestic hot water supply [5].

Solar Water Heating (SWH) is the first and most common application of solar thermal systems, although its application is stagnated by limitations to effective collection and storage of solar energy [6]. SWH technology is important in both the domestic and industrial sectors of many countries as it is a reliable substitute to the conventional fossil fuel, hence leading to savings in energy cost [7, 8]. Mehmood, et al. [7] reports that the solar thermal collector is the main component of a SWH system; it captures the incident solar radiations and converts them to useful energy that heats the water. Research has focused on optimizing the design and performance of solar thermal collectors, including the effects of varying the tilt angle among others. A common challenge to the roll-out of solar water heating systems is the high cost of stand-alone systems, particularly to households. This is a key barrier limiting its broad implementation [8, 9, 10, 11].

Nocturnal radiation cooling is one of the most effective natural passive cooling technologies by infrared radiation exchange between earthly surfaces and the sky [12]. The sun radiates heat to the earth and warms it up during the day, but at night the warmer earth radiates the heat to the sky. The sky at night remains colder than terrestrial surfaces and therefore acts as a heat sink, that is to say, surfaces exposed to the night sky will lose heat by radiation to the sky and in doing so cool down. This phenomenon is termed nocturnal radiative cooling [5, 13, 14, 15, 16, 17, 18, 19, 20, 21, 22, 23]. The importance of harnessing nocturnal cooling resources has been studied by researchers. The governing theories, principles, and applications of night-time radiation cooling as well as assessing possible opportunities and constraints of this ancient yet rarely employed phenomenon have been extensively reviewed [4]. Like most passive processes, nocturnal radiative cooling is dependent upon ambient weather conditions. Yoshihiro and Masato [24] reported that seasonal variations in humidity, cloudiness, and ambient winds affect the intensity of nocturnal cooling. For instance, increased humidity and cloud cover with strong ambient winds exceeding 3.6 m/s will suppress or delay cooling. Bokor, et al. [25] developed a mathematical model to describe the nocturnal cooling process by carrying out building simulations using RETScreen 4 Clean Energy Project Analysis software. The study concluded that locations with drier climates reach higher nocturnal cooling performance since increased moisture content of the atmosphere hampers the radiant heat transfer. The climate of Botswana is semi-arid according to Atekwana, et al. [26], which implies that it is predominantly dry and that theoretically would work to support successful nocturnal radiation cooling according to the findings of Bokor, et al. [25] & Yoshihiro and Masato [24]. Okoronkwo, et al. [27] carried out experimental tests in Owerri, Nigeria to determine the feasibility of using nocturnally cooled water for space cooling. Results showed an average room temperature depression of up to 2.5 °C, which translates to about 101 kJ of cooling energy. Similarly, Nwaigwe [28] carried out transient analysis and performance prediction of passive cooling of a similar system. A mathematical model was developed by transforming differential equations into finite-difference equations for solving the numerical problem. The model predicted a heat removal rate of 57.6 W/m^2^ with a temperature depression of about 1–1.5 °C [28]. Hosseinzadeh and Taherian [2] combined both experimental and numerical assessments of nocturnal radiative cooling using flat plate solar collectors in Iran and achieved up to 7–8 °C water temperature depressions during a clear sky at 0.05 kg/s mass flow rate. The developed mathematical model for a flat-plate solar collector was used as a guideline in deriving the governing equations of a night sky radiator and the solutions of fluid temperature at any position subject to the inlet fluid temperature [2]. Hosseinzadeh and Taherian [2] studied the cooling loop of the storage tank, pump, connecting pipes and the radiator experimentally, and then used the results of the experiments to validate the analytical model for the flat-plate solar collector.

As is the case with solar water heating systems, practical applications of nocturnal radiation cooling systems are not very popular. Deployment of Solar Water Heating Systems (SWHS) and Nocturnal Radiation Cooling Systems (NRCS) as stand-alone systems is cost intensive. Hence a viable approach is a hybrid system that is capable of harnessing solar radiation during the day and night sky radiation, leading to significantly reduced overall cost of investment [5]. The combination of solar water heating and nocturnal radiation cooling improves the economics of the systems in comparison to stand-alone heating and cooling systems [5, 29].

According to Nwaji, et al. [29], hybrid systems are described as systems that have the capability of performing different characteristic functions as a single unit such as generation of solar electricity and heat, solar water heating and space cooling. The shortfalls of stand-alone systems have triggered development of hybrid systems. In a novel study investigating the performance of a hybrid solar water heating and nocturnal water cooling systems, an assessment of the instantaneous hybrid panel performance effects due to bond width, bond thickness, and tube spacing to optimise specifications for the prototype design was undertaken [29]. The parametric analysis showed that the optimum values for bond thickness is 0.003 m, bond width is 0.26m, tube spacing is 0.1m and windscreen requirement is one polyethylene windscreen for convection cover. Further results showed that the maximum temperature attained during diurnal heating was 84.6 °C, while the minimum nocturnal cooling temperature of water attained was 20.21 °C for Owerri. The studies of Nwaji, et al. [5] are based in a tropical region. However, many countries are located outside the tropical region, hence the applicability of the study is limited. Several challenges hamper the development and implementation of solar water heating and nocturnal radiation cooling hybrid systems that will need to be addressed to achieve worldwide implementation success, such challenges include economic, technical, and societal constraints [29, 30, 31, 32, 33, 34].

The application of hybrid panels to countries outside tropical zones motivated the current study. In subtropical regions, the climatic condition is characterized by hot and humid summer conditions, and cold to mild winter conditions. Subtropical regions lie between the tropical belt and temperate regions. The temperate climates experience extreme climatic conditions including lower winter temperatures. The present study presents application of a hybrid panel in a subtropical climate. Many countries lie within the subtropical belt including countries in Africa, Asia, Americas, Europe, Oceania and Southern Indian Ocean. In Africa, over thirty (30) countries fall within this belt including parts of South Africa, Namibia, Zimbabwe, Kenya, and many others. Major parts of Botswana are subtropical, with some areas experiencing arid and semi-arid conditions. A solution that can address the thermal performance of a hybrid panel system suitable for subtropical countries will find relevance and application in many countries, hence the present study presents a case study using Gaborone, Botswana.

## 2 Methodology

**Figure 1:**
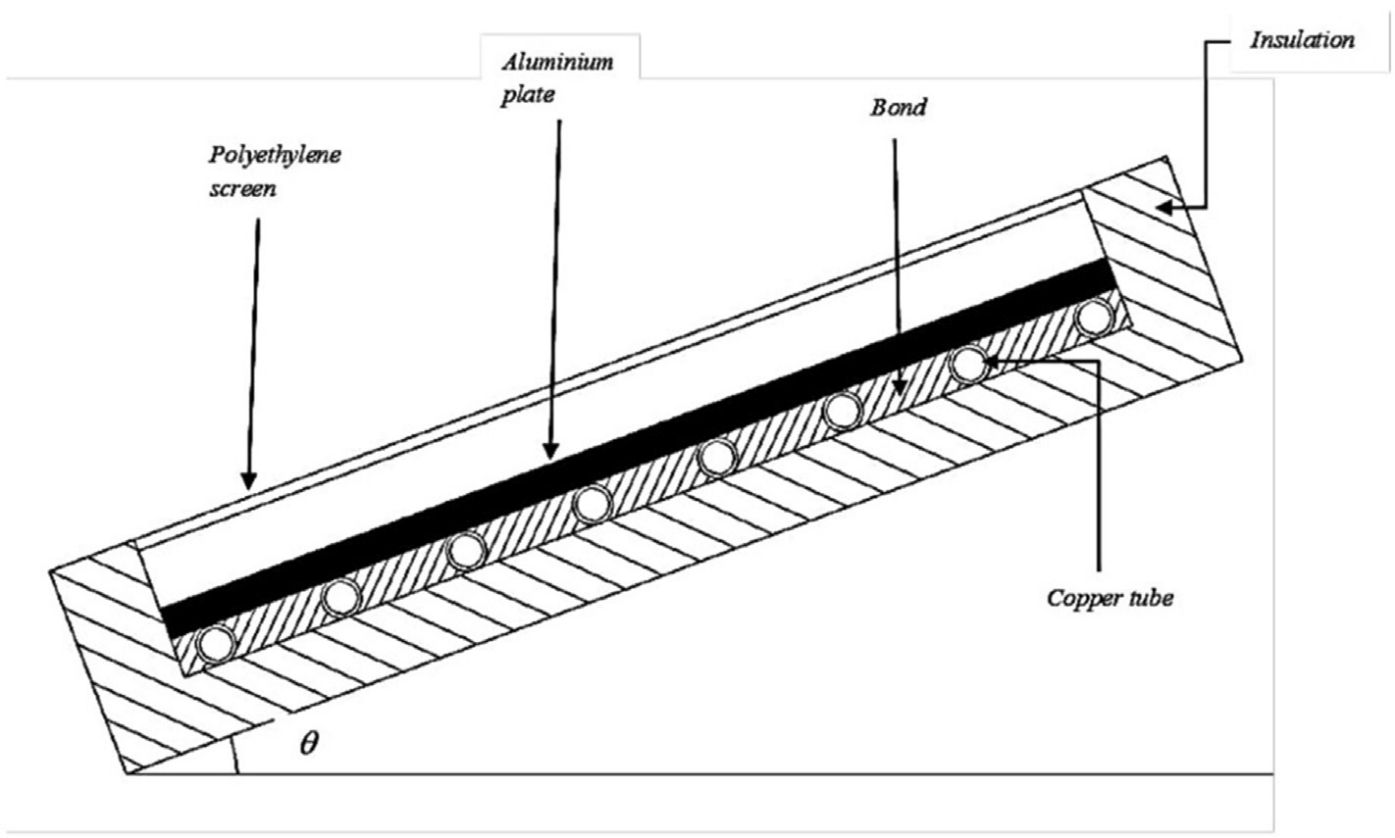
Solar Absorber and Nocturnal radiator panel section.

This study was carried out through the formulation of a thermal mathematical model of a solar absorber and nocturnal radiator (SAANR) panel using the finite difference approach. The solar absorber and nocturnal radiator panel shown in Figure 1 is made of a thin Aluminium plate absorber with copper tubes bonded underneath the plate. Water flows through the copper tubes at a constant flow rate and there is energy transfer between that water and the aluminium plate. The panel was covered by a polyethylene windscreen to minimise convection effects, and it was properly insulated inside its galvanised steel exterior to combat heat transmittance with the ambient environment. A plate surface area of *A*_*s*_ = 18.2m^2^ was selected for the study, to be consistent with Nwaigwe, et al. [28] & Nwaji, et al. [5] who achieved good experimental performance of the panel at that particular surface area.

In analysing the energy transfer on the SAANR panel, the following assumptions were made:

1. The panel is made of thin aluminium plate with a high thermal conductivity, thus resistance to conduction is negligible.
2. A lumped parameter analysis was used, whereby a single temperature is assigned to the whole object. The panel is assumed to have uniform temperature distribution.
3. Axial heat conduction and viscous dissipation of water are neglected.
4. Internal heat generation within the SAANR panel is negligible since the internal resistance of the panel (L/kA) is very small with respect to the convective resistance (1/hA) at the surface.
5. Thermal resistance between the water and the panel surface is relatively small, thus temperature of water in the panel was equal to the panel surface temperature.
6. Mass flowrate of the working fluid (water) remained constant throughout the study.
7. The initial temperature of water was assumed to be constant at a room temperature of 25 °C.
8. The insulation is large enough to stop conduction energy transfer, thus back insulation heat transfer is negligible.
9. During the day sky radiation is offset by solar radiation gains, hence it is negligible.
10. Since polyethylene film has a high spectral transmittance, it is assumed that all of the solar radiation rays are transmitted through the polyethylene screen cover to the absorber surface without being reflected nor refracted.
11. A clear day and clear night sky are assumed for the study.

### 2.1 Nocturnal cooling model

**Figure 2:**
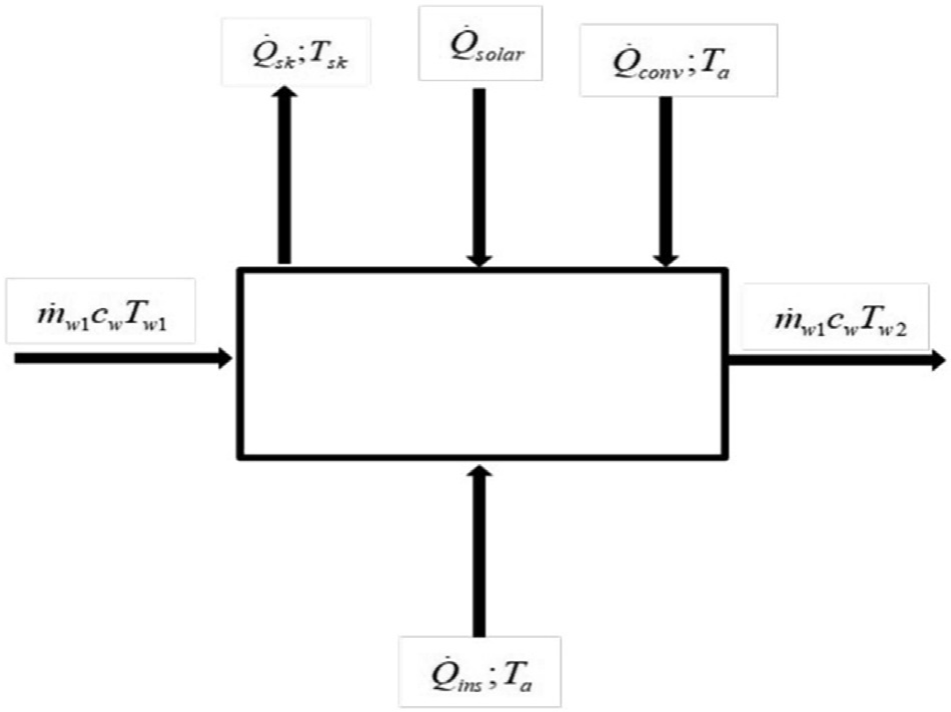
Control volume of the SAANR panel during nocturnal cooling.

Nocturnal radiation cooling is prevalent between the hours of 8 pm and 5 am [28]. Thermally the SAANR panel can be considered as a single control volume of negligible thermal capacitance as in Figure 2.

Using thermodynamics energy balance approach for the control volume:

Energy flow into the SAANR panel (${\dot{Q}}_{in}$) + Energy generated internally (${\dot{Q}}_{i,gen}$) = Energy loss from the SAANR (${\dot{Q}}_{out}$) + Rate of change of Internal energy (H).

(1) $${\dot{Q}}_{in}+{\dot{Q}}_{i,gen}={\dot{Q}}_{out}+H$$

Where;

(2) $$H=mc\left(\frac{\partial T}{\partial t}\right)$$

Forward finite difference scheme was applied to the partial derivative as shown in Eq. (3) [35].

(3) $${\left(\frac{\partial T}{\partial \tau }\right)}_{\xi ,\tau ,fwd}\approx \frac{1}{\partial \tau }\left[T\left(\xi ,\tau +\partial \tau \right)-T\left(\xi ,\tau \right)\right]$$

The partial derivative in linear form with respect to time becomes:

(4) $$\frac{\partial T}{\partial t}=\frac{\left(T^{t+1}-T^{t}\right)}{\Delta t}$$

Thus, the rate of change of internal energy is:

(5) $$H=\left(\frac{m_{t}c_{w}}{\Delta t}\right)\left(T^{t+1}-T^{t}\right)$$

Following the assumption that internal energy generation is negligible, Eq. (1) reduces to:

(6) $${\dot{Q}}_{in}={\dot{Q}}_{out}+H$$

From the control volume, the parameters of the energy balance equation become:

(7) $${\dot{Q}}_{in}={\dot{Q}}_{solar}+{\dot{Q}}_{conv}+{\dot{Q}}_{ins}+{\dot{m}}_{w1}c_{w}T_{w1}$$

(8) $${\dot{Q}}_{out}={\dot{Q}}_{sk}+{\dot{m}}_{w1}c_{w}T_{w2}$$

Energy flow into the control volume consists of, ${\dot{Q}}_{solar}$ = overall solar radiation absorbed by the panel, ${\dot{Q}}_{conv}$ = convective heat transfer between air and the panel, ${\dot{Q}}_{ins}$ = back insulation heat transfer from the ambient air to the panel, and the energy due to water inlet supplied at room temperature.

Energy flow out, however, consists of the ${\dot{Q}}_{sk}$ = panel surface radiation losses to the night sky and the energy due to water outlet from the panel.

The thermal resistance of the insulation material is very high, hence ${\dot{Q}}_{ins}\approx 0$ and can be neglected. Also, during the nocturnal cooling phase, there is little to no solar irradiation (G), hence ${\dot{Q}}_{solar}\approx 0$ and can be neglected as well.

Substituting Eqs. (5), (7), and (8) in Eq. (6) and effecting the assumptions above:

(9) $${\dot{Q}}_{conv}+{\dot{m}}_{w1}c_{w}T_{w1}={\dot{Q}}_{sk}+{\dot{m}}_{w1}c_{w}T_{w2}+\left(\frac{m_{t}c_{w}}{\Delta t}\right)\left(T^{t+1}-T^{t}\right)$$

But;

(10) $$\left(\frac{m_{t}}{\Delta t}\right)={\dot{m}}_{w1}={\dot{m}}_{w}$$

Hence;

(11) $$\left({\dot{m}}_{w}c_{w}\right)\left(T^{t+1}-T^{t}\right)={\dot{Q}}_{conv}+{\dot{m}}_{w}c_{w}\left(T_{w1}-T_{w2}\right)-{\dot{Q}}_{sk}$$

Further rearranging;

(12) $$T^{t+1}=T^{t}+\left(\frac{1}{\left({\dot{m}}_{w}c_{w}\right)}\right)\left[{\dot{Q}}_{conv}+{\dot{m}}_{w}c_{w}\left(T_{w1}-T_{w2}\right)-{\dot{Q}}_{sk}\right]$$

From heat transfer principles, the heat transfer rate between two points is given thus:

(13) $$\dot{Q}=\frac{\left(T_{1}-T_{2}\right)}{R_{th}}$$

Applying Eq. (13) into Eq. (12):

(14) $$T^{t+1}=T^{t}+\left(\frac{1}{\left({\dot{m}}_{w}c_{w}\right)}\right)\left\{\left[\frac{\left(T_{a}-T_{s}\right)}{R_{conv}}\right]-\left[\frac{\left(T_{s}-T_{sk}\right)}{R_{sk}}\right]+\left[{\dot{m}}_{w}c_{w}\left(T_{w1}-T_{w2}\right)\right]\right\}$$

From the assumptions that the thermal capacity of the panel is small, and that thermal resistance between the water and the radiating surface is relatively small, it suggests that the temperature of the water flowing through the panel is approximately equal to the temperature of the panel surface. It is also assumed that the water temperature varies linearly along its flow path since the flow rate is assumed to be steady, thus;

(15) $$T_{s}\approx T_{w}\approx \frac{\left(T_{w1}+T_{w2}\right)}{2}$$

The heat transfer rate from the radiating surface to the sky is:

(16) $${\dot{Q}}_{sk}=\frac{\left(T_{s}-T_{sk}\right)}{R_{sk}}$$

$R_{sk}$ is the thermal resistance to radiation, which is given by the expressions below [36]:

(17) $$R_{sk}=\frac{1}{\left(h_{r}A_{s}\right)}$$

Where the radiative heat transfer coefficient ($h_{r}$) is given below in Absolute temperatures:

(18) $$h_{r}=\frac{\sigma \left(T_{s}^{2}+T_{sk}^{2}\right)\left(T_{s}+T_{sk}\right)}{\frac{1}{\varepsilon_{s}}+\frac{A_{s}}{A_{sk}}\left(\frac{1}{\varepsilon_{sk}}-1\right)}$$

The surface area of the sky ($A_{sk}$) is infinitely big relative to that of the panel, thus;

(19) $$\frac{A_{s}}{A_{sk}}\left(\frac{1}{\varepsilon_{sk}}-1\right)\approx 0$$

Therefore;

(20) $$h_{r}=\varepsilon_{s}\sigma \left(T_{s}^{2}+T_{sk}^{2}\right)\left(T_{s}+T_{sk}\right)$$

$\varepsilon_{s}$ is the emissivity of the panel surface and σ is the Stefan-Boltzmann constant (σ = 5.67∗10^−8^ W/m^2^K^−4^).

According to Meir, et al. [37] and Dobson [36] the sky temperature is approximated relative to the ambient air temperature as:

(21) $$T_{sk}=\varepsilon_{sk}^{0.25}T_{a}$$

$\varepsilon_{sk}$ is the emissivity of the night-sky, which is a function of temperature, relative humidity, and the clearness of the sky.

Convective heat transfer between ambient air and the SAANR surface exposed to the air is:

(22) $${\dot{Q}}_{conv}=\frac{\left(T_{a}-T_{s}\right)}{R_{conv}}$$

Where convection thermal resistance ($R_{conv}$) is given by:

(23) $$R_{conv}=\frac{1}{\left(h_{conv}A_{s}\right)}$$

From Hu, et al. [38] and Nwaji, et al. [5], the convective heat transfer coefficient due to wind for a SAANR panel covered with a polyethylene windscreen ($h_{conv}$) is given by:

(24) $$h_{conv}=0.5+1.2u^{0.5}$$

Where; *u =* average wind velocity.

From Eq. (14) and using solutions from all the other equations discussed above:

(25) $$T^{out}=T^{in}+\left(\frac{1}{{\dot{m}}_{w}c_{w}}\right)\left\{\left[\frac{\left(T_{a}-T_{s}\right)}{R_{conv}}\right]-\left[\frac{\left(T_{s}-T_{sk}\right)}{R_{sk}}\right]+\left[\left({\dot{m}}_{w}c_{w}\right)\left(T^{in}-T^{out}\right)\right]\right\}$$

Simplifying further:

(26) $$\frac{\left(T^{out}-T^{in}\right)}{{\dot{m}}_{w}c_{w}}+\left(T^{out}-T^{in}\right)=\frac{1}{\left({\dot{m}}_{w}c_{w}\right)\left({\dot{m}}_{w}c_{w}\right)}\left\{\left[\frac{\left(T_{a}-T_{s}\right)}{R_{conv}}\right]-\left[\frac{\left(T_{s}-T_{sk}\right)}{R_{sk}}\right]\right\}$$

(27) $$T^{out}=T^{in}+\left(\frac{1}{{\dot{m}}_{w}c_{w}(1+{\dot{m}}_{w}c_{w})}\right)\left\{\left[\frac{\left(T_{a}-T_{s}\right)}{R_{conv}}\right]-\left[\frac{\left(T_{s}-T_{sk}\right)}{R_{sk}}\right]\right\}$$

Eq. (27) is the lumped parameter functional relationship for nocturnal radiation cooling using the SAANR panel.

### 2.2 Diurnal heating model

Diurnal solar heating takes place between the hours of 7 am and 6 pm due to the availability of solar irradiation at that time.

**Figure 3:**
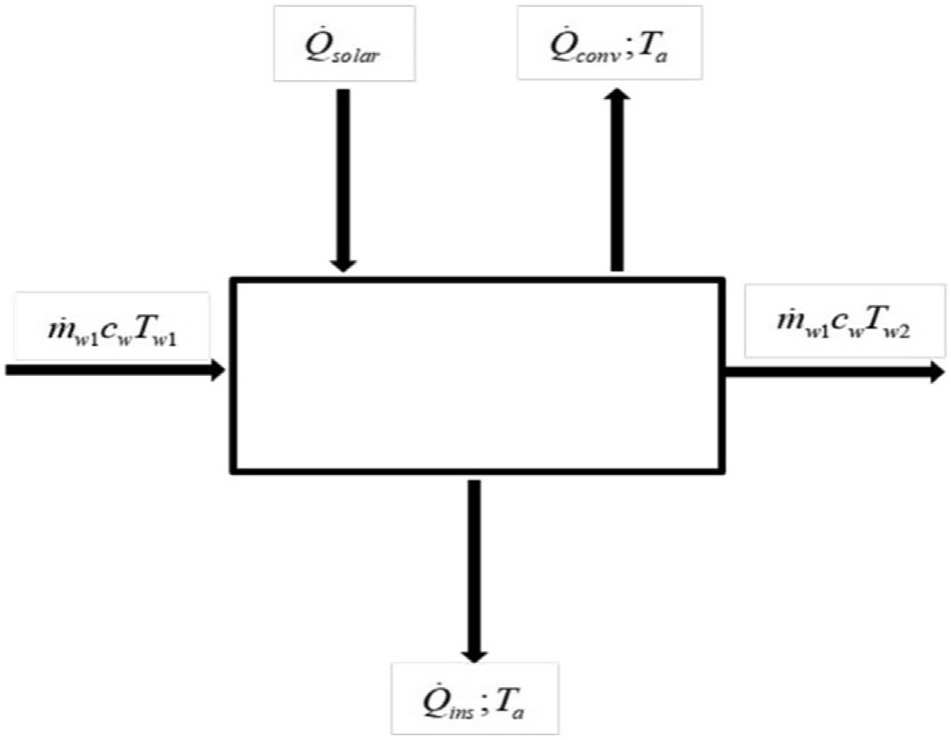
Control volume of the SAANR panel during diurnal heating.

Using thermodynamics energy balance approach for the control volume in Figure 3.

(28) $${\dot{Q}}_{in}+{\dot{Q}}_{i,gen}={\dot{Q}}_{out}+H$$

Rate of change of internal energy after applying forward finite difference scheme as in Eqs. (2), (3), (4), and (5):

(29) $$H=\left({\dot{m}}_{w}c_{w}\right)\left(T^{t+1}-T^{t}\right)$$

Internal energy generation is neglected thus Eq. (28) becomes:

(30) $${\dot{Q}}_{in}={\dot{Q}}_{out}+H$$

From the control volume:

(31) $${\dot{Q}}_{in}={\dot{Q}}_{solar}+{\dot{m}}_{w1}c_{w}T_{w1}$$

(32) $${\dot{Q}}_{out}={\dot{Q}}_{conv}+{\dot{Q}}_{ins}+{\dot{m}}_{w1}c_{w}T_{w1}$$

As in the nocturnal cooling phase, the thermal resistance of the insulation is relatively high thus it is assumed no heat is lost or gained through the insulation material i.e., ${\dot{Q}}_{ins}=0$.

Substituting Eqs. (29), (31), and (32) into Eq. (30):

(33) $${\dot{Q}}_{solar}+{\dot{m}}_{w}c_{w}T_{w1}={\dot{Q}}_{conv}+{\dot{m}}_{w}c_{w}T_{w2}+\left({\dot{m}}_{w}c_{w}\right)\left(T^{t+1}-T^{t}\right)$$

Applying Eq. (13) into Eq. (33):

(34) $$T^{t+1}=T^{t}+\left(\frac{1}{{\dot{m}}_{w}c_{w}}\right)\left\{\left[{\dot{Q}}_{solar}\right]-\left[\frac{\left(T_{s}-T_{a}\right)}{R_{conv}}\right]+\left[{\dot{m}}_{w}c_{w}\left(T_{w1}-T_{w2}\right)\right]\right\}$$

Heat transfer rate due to solar radiation absorbed by the panel surface area:

(35) $${\dot{Q}}_{solar}=\theta \alpha \rho GA_{s}$$

Where; θ is the tilt angle, α is the absorptivity of the surface, ρ is the reflectivity, G is the solar radiation, and *A*_*s*_ is the area of the collector surface.

The term [θαρ] represents a function relating the solar irradiance (*G*) on a horizontal surface to the radiation actually absorbed by the surface, and it is dependent upon the sun position relative to the surface, spectral properties of the radiation and optical surface properties. Its value ranges between 0.43-0.90 [36]. The assumption here is that all of the solar radiation is transmitted through the polyethylene screen. From the studies of Saraf and Hamad [39], Kalogirou [40] and Nwaji, et al. [5], the optimal tilt angle is taken to be equal to the local latitude angle i.e., for Gaborone, Botswana the panel tilt angle will be θ=24.6 ֩.

Convective heat transfer between SAANR surface exposed to air and the ambient air is given by:

(36) $${\dot{Q}}_{conv}=\frac{\left(T_{s}-T_{a}\right)}{R_{conv}}$$

The convection thermal resistance is given in Eq. (23).

Substituting Eq. (35) in Eq. (34) yields;

(37) $$T^{out}=T^{in}+\left(\frac{1}{{\dot{m}}_{w}c_{w}}\right)\left\{\left[\theta \alpha \rho GA_{s}\right]-\left[\frac{\left(T_{s}-T_{a}\right)}{R_{conv}}\right]+\left[{\dot{m}}_{w}c_{w}\left(T^{in}-T^{out}\right)\right]\right\}$$

Simplifying further:

(38) $$\frac{\left(T^{out}-T^{in}\right)}{{\dot{m}}_{w}c_{w}}+\left(T^{out}-T^{in}\right)=\frac{1}{\left({\dot{m}}_{w}c_{w}\right)\left({\dot{m}}_{w}c_{w}\right)}\left\{\left[\theta \alpha \rho GA_{s}\right]-\left[\frac{\left(T_{s}-T_{a}\right)}{R_{conv}}\right]\right\}$$

(39) $$T^{out}=T^{in}+\left(\frac{1}{{\dot{m}}_{w}c_{w}(1+{\dot{m}}_{w}c_{w})}\right)\left\{\left[\theta \alpha \rho GA_{s}\right]-\left[\frac{\left(T_{s}-T_{a}\right)}{R_{conv}}\right]\right\}$$

Eq. (39) is the lumped parameter functional relationship for diurnal solar heating using the SAANR panel.

### 2.3 MATLAB program input parameters

The thermal mathematical models in Eq. (27) and Eq. (39) were then coded in MATLAB R2019b software and simulated. The area of the panel and mass flowrate of water are optimal values adopted from the work of Nwaigwe, et al. [28]. The water at the panel inlet is assumed to be at room temperature approximated at 25 °C. The program input parameters are presented in Table 1.

**Table 1:** Program input parameters.

Parameter	Value
Panel surface area ($A_{s}$)	18.2 m^2^
Mass flowrate ($\dot{m}$)	0.0833 m^3^/s
Absorptivity (α)	0.92
Reflectivity (ρ)	0.04
Tilt angle (θ)	24.6 ֩
Emissivity of surface ($E_{s}$)	0.55
Emissivity of the sky ($E_{sk}$)	0.74
Inlet water temperature ($T_{in}$)	25 °C

## 3 Results and discussion

This study used Gaborone summer and winter weather data harvested by Mutabilwa [41] and Letlhare-Wastic [42] at the University of Botswana. To generate the results the heating model was run from 7am to 6pm, after which the radiation cooling model was to run from 7pm to 6am in the morning of the following day.

**Figure 4:**
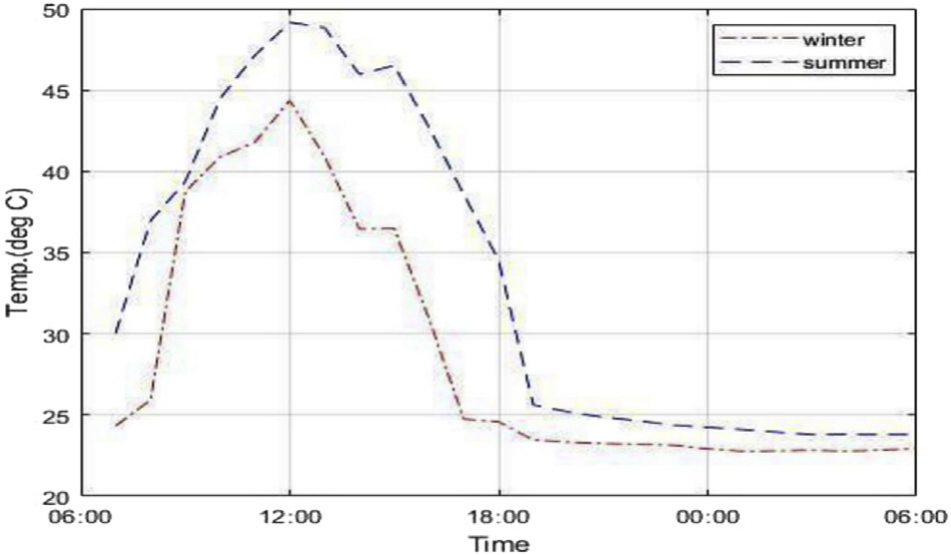
SAANR panel outlet water temperature.

From Figure 4, higher water temperatures are achieved during the day in summer. However, lowest temperatures are yielded for night-time cooling in winter. The solar water heating loop in summer achieved 49 °C maximum water temperatures, having risen the water by 24 °C from room temperature. This summer water temperatures are more than sufficient for water heating purposes. However, one of the key purposes of this study was to determine whether solar water heating can still be applicable in the winter season. In winter the average heated water temperature was 34.2 °C and reached a maximum up to 44.4 °C. This is an increase by 19.4 °C from room temperature inlet water (7 kWh), thus solar water heating can still be effective in winter seasons.

**Figure 5:**
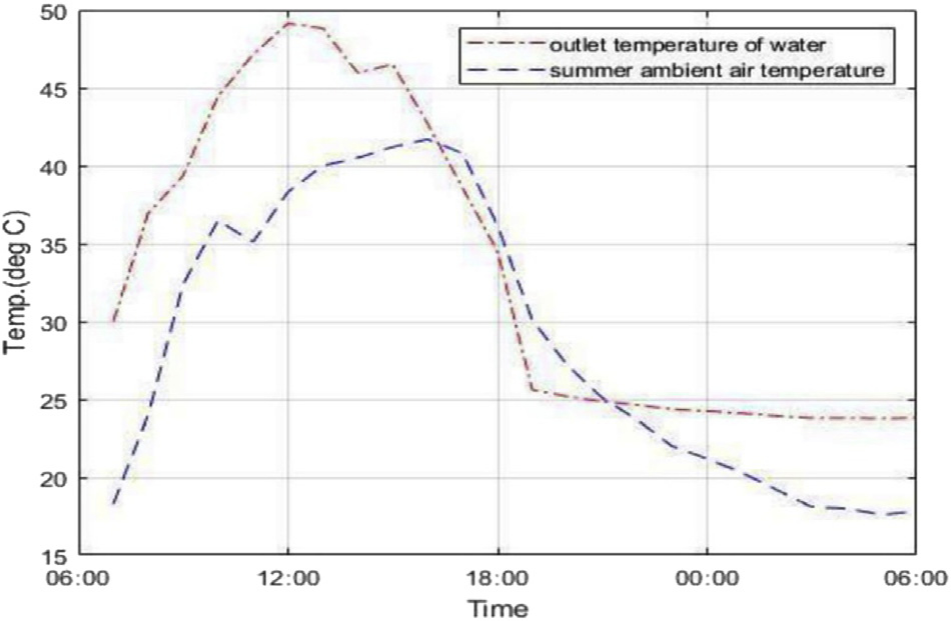
Summer panel outlet and ambient temperatures.

**Figure 6:**
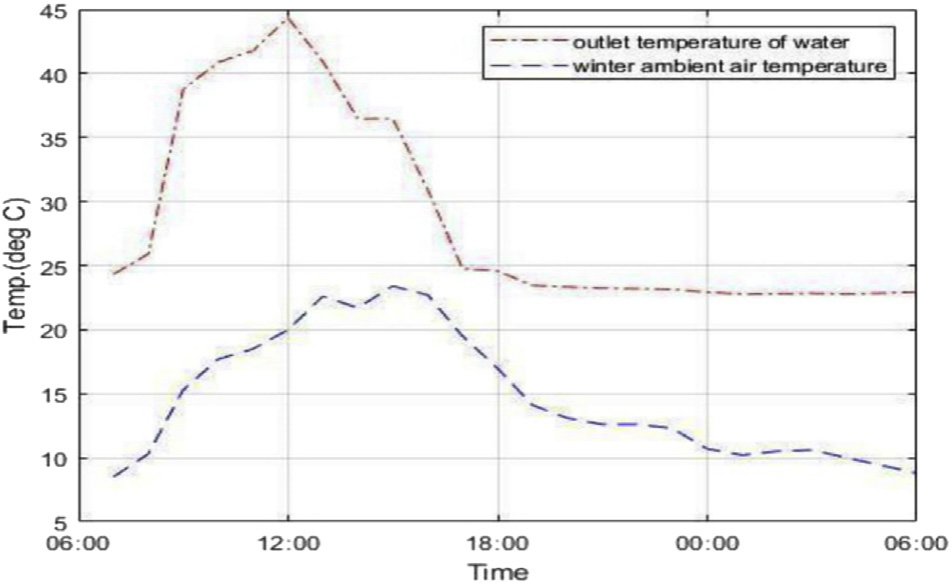
Winter panel outlet and ambient air temperatures.

In the night-time cooling phase, minimum temperatures of 22.8 °C and 23.8 °C were recorded in winter and summer respectively. This corresponds to working fluid temperature drops of 2.2 °C and 1.2 °C in winter and summer respectively. Considering that the average ambient air temperature during the day in summer is around 35.5 °C from Figure 5, water at 23.8 °C can be sufficient to bring about comfort. The amount of energy corresponding to the difference in cooled panel outlet water temperature and inlet water at room temperature in summer is 0.5 kWh. In winter there is no need for space cooling since average ambient temperatures hover around 18 °C during the day. The panel outlet temperature mostly follows the trend of the ambient air environment as presented by Figure 5 and Figure 6, which means ambient air temperature is a key determinant for panel performance.

Figure 5 shows an anomaly in which the outlet temperature of water dropped below the ambient air temperature from around 1600 h until 2200hrs. This is not an expected behaviour of the system and cannot be attributed as a consequence of nocturnal cooling since according to research nocturnal cooling commences at around 2100 h. This result may be a consequence of the assumption of a constant inlet water temperature of 25 °C which may not accurately represent some extremely high summer temperatures in Botswana especially at afternoon hours. Therefore, in reality during summer the room temperatures of water may rise way beyond the assumed figure thus the said figure would not match the recorded ambient environment which would possibly lead to the anomaly depicted in Figure 5.

### 3.1 Validation

The developed model was validated by comparing its panel outlet water temperature with that of experiments carried by Nwaigwe [35] at Owerri, Nigeria on the 10^th^ of August in 2010 during a clear weather day. The statistics software IBM SPSS Statistics Data Editor was used for the statistical analysis in this study. All differences in the descriptive statistics in Table 2 with the exception of the range are within 1 °C, thus an excellent correlation is therefore expected between the two parameters. The regression statistics of model and experimental temperatures are shown in Table 3 for a total of 12 observations, at 95% confidence intervals.

**Table 2:** Descriptive statistics.

Statistic Parameter	T_pre_ (°C)	T_exp_ (°C)	Difference (°C)
Mean	23.02	23.30	0.28
Std. Dev	0.24	0.83	0.59
Min.	22.77	21.80	-0.97
Max.	23.45	24.40	0.95
Range	0.68	2.60	1.92

**Table 3:** Regression statistics.

Regression parameter	Value
Correlation coef. (r)	0.832
R Squared (R^2^)	0.693
Observations (N)	12
Degree of freedom (df = N-2)	10

Correlation describes the strength of association between parameters. It measures the strength and direction of a linear relationship between model and experimental temperatures on a plot. In the case of this study, the correlation coefficient, r = 0.832, which suggests a strong and positive relationship between *T*_*pre*_ and *T*_*exp*_.

The correlation coefficient might be strong and positive, but still there is still a need to test the significance of that correlation coefficient on the data set. From the Pearson correlation coefficient data at two tail proportion and 0.01 significance level for df = 10, r = 0.7079. The correlation of the results is greater than the Pearson critical correlation coefficient, 0.832 > 0.7079, hence the correlation coefficient is considered to be acceptable for significance. In linear regression models, R^2^ is a relative goodness-of-fit measure. The closer the value is to 1, the better the relationship between two parameters. In this study, R^2^ measures the strength of the relationship between model predicted temperatures and experimentally determined temperatures. From Table 3, the R^2^ value is stated as 0.693 which shows a reasonably strong goodness-of-fit, thus low residuals are expected.

## 4 Conclusions

The mathematical models for a SAANR hybrid panel were developed using principles of heat transfer and thermodynamics. The models were developed for a transient system. The resulting equations were transformed into the numerical format using the finite difference scheme, and subsequently used to carry out a transient analysis and performance evaluation of the SAANR panel using a computer program written in MATLAB R2019b. MATLAB software simulations showed that the panel is capable of producing cold water at 23.8 °C in summer, which is about 12 °C less than the average daily ambient air temperatures. According to Gendelis and Jakovics [43], humans generally feel comfortable at envelope temperatures between 22 °C and 24 °C, thus the panel by producing cold water at 23.8 °C can be used for space cooling in summer. Similarly, the SAANR panel is robust enough since it also has favourable results for solar water heating in winter. The panel reaches water temperatures up to 44.4 °C in winter, which is hot enough to produce domestic hot water since studies by Carlos, et al. [44] reported that ideal bath water temperature ranges between 40-45 °C. Overall, this study has discovered that the SAANR hybrid system can be implemented with success in both summer and winter seasons of Gaborone for space cooling and domestic water heating respectively. The model developed in this study was successfully validated by comparison with experimental results from Nwaigwe [35] and showed excellent correlation and a fairly good and acceptable goodness-of-fit.

## Declarations

### Author contribution statement

Kago R. Rabasoma: Conceived and designed the experiments; Performed the experiments; Analyzed and interpreted the data; Contributed reagents, materials, analysis tools or data; Wrote the paper.

Kevin N. Nwaigwe: Conceived and designed the experiments; Analyzed and interpreted the data; Contributed reagents, materials, analysis tools or data; Wrote the paper.

### Funding statement

This research did not receive any specific grant from funding agencies in the public, commercial, or not-for-profit sectors.

### Data availability statement

Data included in article/supp. material/referenced in article.

### Declaration of interests statement

The authors declare no conflict of interest.

### Additional information

No additional information is available for this paper.

## References

[1] Kaynakli O. (2012). A review of the economical and optimum thermal insulation thickness for building applications. Renew. Sustain. Energy Rev..

[2] Hosseinzadeh E., Taherian H. (2012). An experimental and analytical study of a radiative cooling system with unglazed flat plate collectors. Int. J. Green Energy.

[3] Zheng G., Jing Y., Huang H., Gao Y. (2010). Application of improved grey relational projection method to evaluate sustainable building envelope performance. Appl. Energy.

[4] Nwaigwe K.N., Okoronkwo C.A., Ogueke N.V., Anyanwu E.E. (2010). Review of nocturnal cooling systems. Int. J. Energy a Clean Environ. (IJECE).

[5] Nwaji G.N., Okoronkwo C.A., Ogueke N.V., Anyanwu E.E. (2020). Investigation of a hybrid solar collector/nocturnal radiator for water heating/cooling in selected Nigerian cities. Renew. Energy.

[6] Shafieian A., Khiadani M., Nosrati A. (2019). Thermal performance of an evacuated tube heat pipe solar water heating system in cold season. Appl. Therm. Eng..

[7] Mehmood A., Waqas A., Said Z., Rahman S.M.A., Akram M. (2019). Performance evaluation of solar water heating system with heat pipe evacuated tubes provided with natural gas backup. Energy Rep..

[8] Abdullahi D., Suresh S., Renukappa S., Oloke D. (2017). Key barriers to the implementation of solar energy in Nigeria: a critical analysis. IOP Conf. Ser. Earth Environ. Sci..

[9] Shaaban M., Petinrin J.O. (2014). Renewable energy potentials in Nigeria: meeting rural energy needs. Renew. Sustain. Energy Rev..

[10] Emodi N.V., Boo K.J. (2015). Sustainable energy development in Nigeria: current status and policy options. Renew. Sustain. Energy Rev..

[11] Charles A. (2014). How is 100% renewable energy possible in Nigeria?. Glob. Energy Netw. Instit. (GENI).

[12] Man Y., Yang H., Qu Y., Fang Z. (2015). A novel nocturnal cooling radiator used for supplemental heat sink of active cooling system. Procedia Eng..

[13] Cheruy F., Dufresne J.L., Aït Mesbah S., Grandpeix J.Y., Wang F. (2017). Role of soil thermal inertia in surface temperature and soil moisture-temperature feedback. J. Adv. Model. Earth Syst..

[14] Chow W.T.L., Pope R.L., Martin C.A., Brazel A.J. (2010). Observing and modeling the nocturnal park cool island of an arid city: horizontal and vertical impacts. Theor. Appl. Climatol..

[15] Family R., Mengüç M.P. (2017). Materials for radiative cooling: a review. Procedia Environ. Sci..

[16] French A.J., Parker M.D. (2010). The response of simulated nocturnal convective systems to a developing low-level jet. J. Atmos. Sci..

[17] Hollick J. (2012). Nocturnal radiation cooling tests. Energy Procedia.

[18] Imran A.A., Jalil J.M., Ahmed S.T. (2015). Induced flow for ventilation and cooling by a solar chimney. Renew. Energy.

[19] Konarska J., Holmer B., Lindberg F., Thorsson S. (2016). Influence of vegetation and building geometry on the spatial variations of air temperature and cooling rates in a high-latitude city. Int. J. Climatol..

[20] Leconte F., Bouyer J., Claverie R., Pétrissans M. (2016). Analysis of nocturnal air temperature in districts using mobile measurements and a cooling indicator. Theor. Appl. Climatol..

[21] Martínez D., Jiménez M.A., Cuxart J., Mahrt L. (2010). Heterogeneous nocturnal cooling in a large basin under very stable conditions. Boundary-Layer Meteorol..

[22] Sima J., Sikula O., Kosutova K., Plasek J. (2014). Theoretical evaluation of night sky cooling in the Czech republic. Energy Procedia.

[23] Wang Z.H., Li Q. (Apr 2017). Thermodynamic characterisation of urban nocturnal cooling. Heliyon.

[24] Yoshihiro I., Masato L. (2002). The influence of seasonally varying atmospheric characteristics on the intensity of nocturnal cooling in a high mountain hollow. J. Appl. Meteorol..

[25] Bokor B., Kajtár L., Eryener D. (2017). Nocturnal radiation: new opportunity in building cooling. Energy Procedia.

[26] Atekwana E.A., Molwalefhe L., Kgaodi O., Cruse A.M. (2016). Effect of evapotranspiration on dissolved inorganic carbon and stable carbon isotopic evolution in rivers in semi-arid climates: the Okavango Delta in North West Botswana. J. Hydrol.: Reg. Stud..

[27] Okoronkwo C.A., Nwigwe K.N., Ogueke N.V., Anyanwu E.E., Onyejekwe D.C., Ugwuoke P.E. (2014). An experimental investigation of the passive cooling of a building using nighttime radiant cooling. Int. J. Green Energy.

[28] Nwaigwe K.N., Okoronkwo C.A., Ogueke N.V., Ugwuoke P.E., Anyanwu E.E. (2012). Transient analysis and performance prediction of nocturnal radiative cooling of a building in Owerri,Nigeria. Res. J. Appl. Sci. Eng. Technol..

[29] Nwaji G.N., Okoronkwo C.A., Ogueke N.V., Anyanwu E.E. (2019). Hybrid solar water heating/nocturnal radiation cooling system I: a review of the progress, prospects and challenges. Energy Build..

[30] Ogueke N.V., Anyanwu E.E., Ekechukwu O.V. (2009). A review of solar water heating systems. J.Renew. Sustain. Energy Rev..

[31] Veeraboina P., Yesuratnam G. (2014). Optimal design & analysis of solar water heating system using solar factors for energy efficiency & thermal performance. Univ. J . Renew. Energy.

[32] Rostamzadeh A., Adim M., Sabzi S. (2011). Experimental and numerical study of forced circulation solar water heaters. Global Conference on Global Warming.

[33] Kariuki D. (2018). Barriers to renewable energy technologies development.

[34] Mursan I.C., Chiciudean G.O., Harun R., Arion F.H., Porutiu A., Chiciudean D.I. (2017). Constraints on use of renewable energy technologies in the rural area: a case study from the north-west region of Romania. Environ. Prot. Ecol..

[35] Nwaigwe K.N. (2011). Transient analysis and performance prediction of passive cooling of buildings using long wave nocturnal radiation in Owerri, Nigeria. PhD, Postgraduate School.

[36] Dobson R.T. (2005). Thermal modelling of a night sky radiation cooling system. Energy South Afr..

[37] Meir M.G., Rekstad J.B., LØvvik O.M. (2002). A study of a polymer-based radiative cooling system. Sol. Energy.

[38] Hu M., Pei G., Li L., Zheng R., Li J., Ji J. (2015). Theoretical and experimental study of spectral selectivity surface for both solar heating and radiative cooling. Int. J. Photoenergy.

[39] Saraf G.R., Hamad F.A.W. (1988). Optimum tilt angle for a flat plate solar collector. Energy Convers. Manag..

[40] Kalogirou S. (2009). Solar Energy Engineering: Processes and Systems.

[41] Mutabilwa P. (2019). Experimental and Numerical Evaluation of a Solar Dryer for Drying Banana.

[42] Letlhare-Wastic K. (2019). A Model that Predicts Indoor Temperature within a Traditional Adobe Mud Hut; Botswana Climatic Conditions.

[43] Gendelis S., Jakovics A. (2017). Thermal comfort condition assessment in test buildings with different heating/cooling systems and wall envelopes. Energy Procedia.

[44] Carlos F., Luis K., Pedro M., Nidia C. (2018). Water consumption monitoring system for public bathing facilities. Energy Procedia.

